# Identification of Material Constants for Composite Materials Using a Sensitivity-Based Multi-Level Optimization Method

**DOI:** 10.3390/ma18122737

**Published:** 2025-06-11

**Authors:** Ching Wen Liu, Tai Yan Kam

**Affiliations:** Department of Mechanical Engineering, National Yang Ming Chiao Tung University, Hsin Chu 30010, China; a72113009@gmail.com

**Keywords:** composite materials, composite plate, identification of material constants, optimization, free vibration, natural frequency

## Abstract

Composite materials have been widely used to fabricate highly reliable composite structures. Since the material constants of the composite structures are important parameters for the reliability assessment of the structures, it is thus desired to have an efficient and effective technique to determine the actual material constants of the constituent materials. In this paper, a novel sensitivity-based multi-level optimization method, which is composed of several level-wise optimization stages, is presented to identify the actual material constants of structures using measured natural frequencies. In the proposed method, the natural frequency sensitivity information for a structure is used to establish the objective functions and conduct the selection of appropriate design variables at different optimization levels. In each level-wise optimization, the number of design variables is properly reduced to simplify the optimization so that the solution can be attained easily and efficiently. The solutions of the level-wise optimization problems produce the expected values and coefficients of variation for the estimates of the material constants. An acceptance criterion established on the basis of the coefficient of variation has been used to assist the identification of the actual material constants. The accuracy verification and applications of the proposed method have been demonstrated by means of several numerical and experimental examples on the identification of material constants for composite plates with different lamination arrangements.

## 1. Introduction

A structure operating in a severe environment may experience material degradation which can weaken the structural parameters such as the stiffness or material constants of the structure. If appropriate measures have not been taken, the progressive weakening of the structural parameters may eventually lead to the failure of the structure. To prevent an unexpected structural failure from occurring, the health of a structure is required to be assessed at appropriate points in time during the lifetime of the structure. Furthermore, it is also desired to have a simple technique that can determine the actual material constants of the structure in an efficient and effective way. Regarding structural health assessment, many researchers have proposed identification methods in which various measured structural responses, such as displacements/strains [1,2,3], wave propagation characteristics [4,5,6,7], ultrasonic scanning [8,9], vibration data [10,11,12,13,14,15,16,17,18,19,20,21,22,23,24,25,26,27,28,29,30,31,32], etc., have been used to identify the actual material constants or structural damages for different types of structures. In particular, the modal characteristics (natural frequencies and mode shape) extracted from vibration data have found applications for material constants identification of composite plate structures. For instance, De Wilde [25], Lai and Lau [27], Moussu and Nivoit [28], and Pedersen and Frederiksen [30] have used experimental and theoretical eigenfrequencies to identify the four material constants (E_1_ E_2_, G_12_, ν_12_) of thin laminated composite plates. In previous studies, however, no attention has been paid to the determination of the through-thickness shear modulus (*G*_23_), which may have significant effects on the vibration of relatively thick composite plates. A number of researchers have proposed methods to determine the through-thickness shear modulus [33,34,35]. The previous methods, however, are complex or tedious in use for determining *G*_23_. On the other hand, as reported in the literature, optimization methods have been used in the design or parameters identification of structural systems. It is noted that the number of design variables has great effects on the attainment of the true solution for an optimization problem or the convergence of the solution. In general, the attainment of the true solution becomes more difficult as the number of the design variables gets larger. Hence, to circumvent this difficulty, the multi-level optimization method has been used to solve complex structural design or system parameters identification problems using different kinds of measured structural responses [36,37,38,39,40,41,42,43,44,45,46,47,48,49,50,51,52]. The basic idea of the multi-level optimization method is to convert the initial optimization problem of a complex system to a number of simple level-wise optimization problems which are solved sequentially to update the values of the design variables until the actual values have been obtained. In general, when the number of design variables adopted to formulate the level-wise optimization problem is properly reduced, the optimization process will be significantly simplified so that the search for the level-wise solution will become more efficient and effective. Hence, the solutions of a set of hierarchically structured level-wise optimization problems will finally produce the actual values for all the design variables. Regarding the identification of material constants for laminated composite plates, the multi-level optimization method may be a useful tool to determine the five material constants, including *G*_23_, of the composite plates.

In this paper, a simple yet effective multi-level optimization method is presented to identify the material constants of composite plates using the theoretically and experimentally predicted natural frequencies. The sensitivity analysis of natural frequency with respect to material constants is performed to study the effects of material constants on the natural frequency sensitivity of the composite plates. The sensitivity information is used to help construct each level-wise optimization problem in which the sum of the differences between specifically chosen theoretical and experimental natural frequencies is used to establish the level-wise objective function. The solution of the level-wise minimization problem is achieved by updating a reduced number of material constants via the use of a global minimization technique. The material constants identifications of rectangular cantilever composite plates with different lamination arrangements and length-to-thickness ratios are used to verify the accuracy and illustrate the applications of the proposed method.

## 2. Formulation of the Sensitivity-Based Multi-Level Optimization Method

The use of measured natural frequencies for the identification of material constants for a composite plate is usually formulated as the following constrained optimization problem.

(1) $$\begin{array}{c} \text{Minimize:}\\ e\left(x\right)=\sum_{j=1}^{N_{F}}{\left(\frac{f_{j}^{*}-f_{j}}{f_{j}^{*}}\right)}^{2}\xi_{j}\\ \text{Subject to:}\\ x_{i}^{L}\leq x_{i}<x_{i}^{U},i=1,\ldots ,N_{C} \end{array}$$

where *e*(***x***) is the objective function measuring the sum of the square of the differences between the predicted and measured natural frequencies; ***x*** = [*E*_1_, *E*_2_, *G*_12_, *ν*_12_, *G*_23_] the elastic constants with *x*_1_ = *E*_1_, *x*_2_ = *E*_2_, *x*_3_ = *G*_12_, and *x*_4_ = *ν*_12_, and *x*_5_ = *G*_23_; *x_i_^L^* and *x_i_^U^* are, respectively, the lower and upper bounds of the elastic constants *x_i_*; *N_C_* is number of material constants; *ξ_j_* (j = 1,…, *N_F_*) are weighting factors used to make the natural frequencies have appropriate contributions to the objective function and avoid the occurrence of numerical underflow of the objective function; and *N_F_* is number of measured natural frequencies. It is noted that when *G*_23_ is included in the identification process, the direct solution of Equation (1) using the conventional optimization algorithms may encounter the convergence problem or even have some difficulty in obtaining the correct solution.

The natural frequencies of a composite plate with given dimensions depend on the magnitudes of the constituent material constants. In the sensitivity analysis of the plate natural frequencies with respect to the material constants, it has been realized that some of the material constants may have larger effects than the others, which may even have no effects on the natural frequencies. Thus, if such natural frequency sensitivity information is properly used in the material constants identification process, the search for the actual material constants may be greatly expedited. Herein, a sensitivity-based multi-level optimization method that involves the solutions of a number of level-wise optimization problems is presented to identify the material constants of composite plates. The universal set of *N_f_* pairs of theoretical and experimental natural frequencies are defined as **FT**, in which the frequencies in each pair have the same modal number. Based on the natural frequency sensitivity information, the natural frequency universal set is further divided into *F_N_* natural frequency subsets **FS_i_** (i = 1,…, *F_N_*), which may not be mutually exclusive, i.e., some natural frequencies may exist in several natural frequency subsets. The number of the natural frequency pairs in any natural frequency subset may be less than or equal to *N_f_*. On the other hand, the universal set of material constants **CT**, containing the *Nc* material constants is divided into *C_N_* material constant subsets **SC_i_** (i = 1, …, *C_N_*), which may not be mutually exclusive, i.e., some material constants may exist in other material constant subsets. It is noted that the number of material constants in any material constant subset may be less than or equal to *Nc*. It is noted that the number of design variables may be greatly reduced so that the solution of the level-wise optimization problem may be easily accomplished. On the other hand, the sequential solutions of the level-wise optimization problems will produce the expected values and coefficients of variation (COVs) for all the material constants. It is noted that when the COV of a material constant estimate is less than 1%, the differences between the expected and actual values of the material constants will be so small that, from the engineering point of view, the expected value can be treated as the actual value of the material constant. If the COV of a material constant estimate is larger than 1%, it can be used to set up the lower and upper bounds on the material constants. On the other hand, the expected values of the specific material constants obtained in the previous levels of optimization will be kept unchanged when solving the current level-wise optimization problem. It will be shown that the solutions of a number of properly selected level-wise optimization problems will produce the best estimates of all the actual material constants. First, consider the first-level optimization problem, in which, for instance, the natural frequencies in natural frequency subset **FS_k_** are highly sensitive to the material constants in material constant subset **CS_i_**. The level-wise optimization problem is then expressed as follows:

(2) $$\begin{array}{c} \text{Minimize:}\\ e\left({CS}_{i}\right)=\sum_{j=1}^{N_{P_{k}}}\left[{\left(\frac{f_{j}-f_{j}^{*}}{f_{j}^{*}}\right)}^{2}\right]\xi_{j}\;\;\;\;\;\;\;\;\;\;\;\;\\ \text{Subject to:}\\ {C_{\mathrm{ir}}}^{L}\;<\;C_{\mathrm{ir}}\;<{C_{\mathrm{ir}}}^{U}\;\;\;\;\;\;\;\;\;\;\;\;r\;=\;1,\ldots ,\;M_{C_{i}} \end{array}$$

where $N_{P_{k}}$ is the number of theoretical (or measured) natural frequencies in natural frequency subset **FS_k_**; C_ir_^L^, C_ir_^U^ are lower and upper bounds of material constant C_ir_ in material constant subset **CS_i_**; and *M_Ci_* is number of material constants in subset **CS_i_**. It is noted that only the material constants in **CS_i_** are treated as the design variables while the original values of the other material constants in **CT** are kept unchanged when solving the above level-wise optimization problem. The use of the reduced number of design variables (material constants) in formulating the above optimization problem can attain the solution easily and produce good estimates of the chosen design variables as well. Here, in this level of optimization, a multi-start optimization technique in which several starting points are randomly generated is adopted to solve the optimization problem by producing the expected values and COVs of the design variables. In the second level of optimization, another material constant subset together with the associated natural frequency subset is used in Equation (2) for material constants identification. It is noted that only the chosen material constants which have not been updated are treated as design variables at this level of optimization. After all the material constants have been updated through a series of level-wise optimizations, the last level-wise optimization is then performed to search for the true values of the design variables with COVs larger than 1%. In the last level-wise optimization problem, all the selected natural frequencies are used to establish the objective function and the COVs (or standard deviations) to set up the lower and upper bounds on the material constants with COVs larger than 1% is established. Hence, at the final stage, the last level-wise optimization problem can be stated as follows:

(3) $$\begin{array}{c} \text{Minimize:}\\ e\left(CT\right)=\sum_{j=1}^{N_{f}}\left[{\left(\frac{f_{j}-f_{j}^{*}}{f_{j}^{*}}\right)}^{2}\right]\xi_{j}\\ \text{Subject to:}\\ {x_{i}}^{L}\;<\;x_{i}\;<\;{x_{i}}^{U}\;\;\;\;\;\;\;\;\;\;\;\;i\;=\;1,2,\ldots ,\;N_{V} \end{array}$$

where *N_V_* is the number of material constants with COVs larger than 1%. Before proceeding to the solution of any of the above level-wise optimization problems for material constants identification, the constrained minimization problems stated in Equations (2) and (3) are first converted to the following unconstrained minimization problem by introducing the general augmented Lagrangian [53].

(4) $$\overset{¯}{\psi }\left(x,\alpha ,\eta ,r_{p}\right)=e\left(x\right)+\sum_{j=1}^{N}\left[\alpha_{j}z_{j}+r_{p}{z_{j}}^{2}+\eta_{j}\phi_{j}+r_{p}{\phi_{j}}^{2}\right]$$

(5) $$\begin{array}{c} z_{j}=max\left[g_{j}\left(x_{j}\right),\frac{-\alpha_{j}}{2\gamma_{p}}\right]\\ g_{j}\left(x_{j}\right)=x_{j}-{x_{j}}^{U}\leq 0\\ \phi_{j}=max\left[H_{j}\left(x_{j}\right),\frac{-\eta_{j}}{2\gamma_{p}}\right]\\ H_{j}\left(x_{j}\right)={x_{j}}^{L}-x_{j}\leq 0;j=1,2,\cdots N \end{array}$$

where *N* is number of constraints; *α_j_*, *η_j_*, *r_p_* are multipliers; and max [*,*] takes on the maximum value of the numbers in the bracket. The updated formulas for the multipliers *α_j_*, *η_j_*, and *r_p_* are as follows:

(6) $$\begin{array}{c} {\alpha_{j}}^{n+1}={\alpha_{j}}^{n}+2{\gamma_{p}}^{n}{z_{j}}^{n}\\ {\eta_{j}}^{n+1}={\eta_{j}}^{n}+2{\gamma_{p}}^{n}{\phi_{i}}^{n};j=1,2,\cdots N\\ {\gamma_{p}}^{n+1}=\left\{\begin{array}{ccc} \gamma_{0}{\gamma_{p}}^{n} & if & {\gamma_{p}}^{n+1}<{\gamma_{p}}^{max}\\ {\gamma_{p}}^{max} & if & {\gamma_{p}}^{n+1}\geq {\gamma_{p}}^{max} \end{array}\right. \end{array}$$

where the superscript *n* denotes iteration number; *γ*_0_ is a constant; and *r_p_*^max^ is the maximum value of *r_p_*. Following the guideline given in the literature [53], the parameters *μ_j_*^0^, η*_j_*^0^, *r _p_*^0^, *γ*_0_, and *r*_p_^max^ are chosen as follows:

(7) $$\begin{array}{c} {\alpha_{i}}^{0}=1.0,{\eta_{i}}^{0}=1.0,j=1,2,\cdots N\\ \gamma_{0}=2.5,{\gamma_{p}}^{0}=0.4,{\gamma_{p}}^{max}=100 \end{array}$$

It is noted that each level-wise optimization problem is first converted to the above unconstrained optimization problem which can be solved using an appropriate optimization technique. Herein, the Multi-Trajectories Optimization Method (MTOM) [54] is adopted to solve the unconstrained optimization problem. In the adopted optimization algorithm, the objective function is treated as the potential energy of a traveling particle and a number of starting points are randomly generated in the feasible region. The idea of the adopted optimization algorithm can be explained using an example of the motion of a mass particle rolling down from any point on a hill. Hence, for a given starting point, when the particle starts rolling down the hill, the equation of motion of the particle in a conservative force field is used to establish a search trajectory. Along the search trajectory, a number of local minima, including the lowest local minimum, will be attained. It is noted that through a series of numerical tests, it has been found that the lowest local minimum is generally in the vicinity of the global minimum. In addition, the lowest local minimum produces the possible outcomes of the design variables (material constants). Consequently, the randomly generated starting points will produce a set of lowest local minima which can be used to assess the statistics of the design variables. The statistics, namely, the expected values, μ*_i_*, standard deviations, σ*_i_*, and COVs of the possible outcomes will be used to identify the actual material constants or update the lower and upper bounds on the material constants. It is noted that the expected values of the design variables obtained at the current level-wise optimization stage will be kept constant when solving the subsequent level-wise optimization problems. On the other hand, at the final stage, if the COVs of some material constants are larger than 1%, the last level-wise optimization problem will be formulated in such a way that only those with COVs larger than 1% are treated as design variables and all the natural frequency pairs in **FT** used to construct the objective function. Furthermore, the standard deviations of the design variables are used to set up the lower and upper bounds on the design variables. The last level-wise optimization problem is again solved using the MTOM to search for the best estimates of the actual values of the design variables. Regarding the solution of a level-wise optimization problem, during the optimization process, the search direction must avoid being dominated by certain material constants, of which the gradient components can cause some difficulty for the solution to converge. Hence, the search direction must be modified in a proper way so that all the design variables will have appropriate contributions to the search direction, which can then lead to the lowest local minimum easily.

Therefore, each gradient component *GD_i_* is modified by introducing the weighting factor *GDW_i_* to establish the modified gradient *MGD_i_*.

$${MGD}_{i}={GDW}_{i}•{GD}_{i}$$

with

(8) $${GDW}_{i}=\frac{{LM}_{max}}{{LM}_{i}}$$

where *LM_i_* is the degree of sensitivity for the *i*th design variable and *LM_max_* the largest degree of sensitivity among the design variables. Here, the degrees of sensitivity are obtained in the frequency sensitivity analysis. During the search process, the updated value of the *i*th design variable, x_i_*, is then obtained as follows:

(9) $${x_{i}}^{*}=x_{i}+D_{t}\frac{{MGD}_{i}}{\sqrt{\sum {\left({MGD}_{i}\right)}^{2}}}$$

where *D_t_* is the selected increment size. It is noted that the choice of the value of *D_t_* can also affect the convergence of the solution. Herein, the magnitude of the objective function is used as a criterion to choose the value of *D_t_*. In general, *D_t_* is selected in such a way that *D_t_* = 0.25, 0.15, and 0.15/*n* with *n* equal to iteration number, respectively, for the objective function being larger than or equal to 0.1, in the interval [0.1, 0.01], and smaller than 0.01, i.e., in the vicinity of the local minimum.

## 3. Natural Frequency Sensitivity Analysis of the Composite Plate

**Figure 1:**
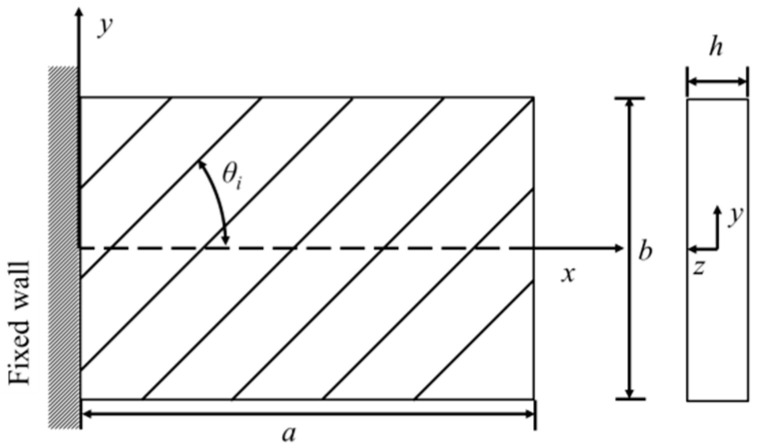
Dimensions of the composite cantilever plate.

The natural frequencies of a composite structure are important system parameters that may contain rich information about the properties, as well as the integrity, of the structure. For illustration, the cantilever composite plate shown in Figure 1 is used to illustrate the applications of the proposed multi-level optimization method for the identification of material constants. Regarding the free vibration analysis of composite plates, the semi-analytical [55] or finite element methods [56] will be used to determine the natural frequencies of the plates. Regarding the rectangular symmetrically laminated cantilever plate under consideration, the plate size is *a* (length) × *b* (width) × *h* (thickness) and the fiber angle of the *i*th layer in the laminate is *θ_i_*. The *x*-*y* plane of the reference coordinate system *x*-*y*-*z* is located at the mid-plane of the plate with 0 ≤ *x* ≤ *a* and − *b*/2 ≤ *y* ≤ *b*/2.

In the adopted semi-analytical method, the displacement field of the laminated plate is written as follows:

(10) $$\begin{array}{c} u\left(x,y,z,t\right)=-z\frac{\partial w_{b}}{\partial x};\\ v\left(x,y,z,t\right)=-z\frac{\partial w_{b}}{\partial y};\\ w\left(x,y,z,t\right)=\left[w_{b}\left(x,y,t\right)+w_{s}\left(x,y,t\right)\right] \end{array}$$

where *u*, *v*, and *w* are the displacement components in the *x*-*y*-*z* coordinate system of the plate; *w_b_* is the vertical deflection induced by bending; and *w_s_* is the vertical deflection induced by through thickness shear deformation. In the free vibration analysis, the vertical displacement components of the plate are expressed as follows:

(11) $$\begin{array}{c} w_{b}\left(x,y,t\right)=W_{b}\left(x,y\right)\sin\omega t;\\ w_{s}\left(x,y,t\right)=W_{s}\left(x,y\right)\sin\omega t \end{array}$$

where *W_b_* and *W_s_* are the deflected shapes induced by bending and through thickness shear deformation, respectively. Discarding the effect of time, the strain-displacement relations of the plate are expressed as follows:

(12) $$\begin{array}{c} \varepsilon_{x}=-z\frac{\partial^{2}W_{b}}{{\partial x}^{2}}\\ \varepsilon_{y}=-z\frac{\partial^{2}W_{b}}{{\partial y}^{2}}\\ \gamma_{xy}=-z\frac{\partial^{2}W_{b}}{\partial x\partial y}\\ \gamma_{yz}=\frac{\partial W_{s}}{\partial y}\\ \gamma_{xz}=\frac{\partial W_{s}}{\partial x} \end{array}$$

where *ε* and *γ* are the normal and shear strains, respectively. The stress–strain relations of a composite lamina with arbitrary fiber angle can be expressed in the following general matrix form [57]:

(13) $$\sigma_{i}={\bar{Q}}_{i}\varepsilon_{i}$$

where ***σ*** and ***ε*** are vectors containing the stresses and strains, respectively and ${\bar{Q}}_{i}$ is the matrix containing the transformed lamina stiffness coefficient, which is dependent on the material constants (*E*_1_, *E*_2_, *ν*_12_, *G*_12_, *G*_23_) and lamina fiber angle *θ_i_*. Here, the determination of the modal characteristics (deflected shape and natural frequency) is achieved via the use of the Ritz method and Hamilton’s principle. In utilizing the Ritz method, two independent sets of characteristic functions have been chosen to approximate *W*_b_ and *W*_s_, which can satisfy the boundary conditions at the fixed edge.

(14) $$W_{b}=\sum_{i=2}^{M_{b}}\sum_{j=0}^{N_{b}}A_{ij}x^{i}y^{j}$$

and

(15) $$W_{s}=\sum_{i=1}^{M_{s}}\left\{B_{i00}\sin\left(\frac{i\pi x}{2}\right)+\sum_{j=1}^{N_{s}}\left[B_{ij1}\sin\left(\frac{i\pi x}{2}\right)\sin\left(j\pi y\right)+B_{ij2}\sin\left(\frac{i\pi x}{2}\right)\cos\left(j\pi y\right)\right]\right\}$$

where *A_ij_* and *B_ijk_* are unknown constants; *M_b_*, *N_b_*, *M_s_*, *N_s_* are numbers of terms in the characteristic functions; and *k* is 0, 1 or 2.

According to Hamilton’s principle, *W*_b_ and *W*_s_ can be used to determine the plate maximum kinetic and strain energies for establishing the Lagrangian, which is the difference between the maximum kinetic and strain energies of the plate. The extremization of the Lagrangian leads to the following eigenvalue problem.

[***K*** − ***ω***^2^ ***M***] ***C*** = 0 (16)

where ***K*** is the stiffness matrix, ***M***, the mass matrix, and ***C*** the vector containing the undetermined constants in Equations (14) and (15). The solution of the above eigenvalue problem can produce the natural frequencies and mode shapes for the plate. It has been shown that *M_b_* = *N_b_* = 10 for *W_b_* and *M_s_* = *N_s_* = 5 for *W*_s_ can produce good predictions of the first five natural frequencies for thin, as well as relatively thick, composite plates. On the other hand, in the adopted finite element method, the SHELL181 elements in the finite element code of ANSYS 19.5 are used to determine the natural frequencies of the plate. Both the semi-analytical and finite element method can produce the same natural frequencies for the plate. It is noted that the natural frequencies are dependent on the material constants of the plate. Once the degradation of the plate material occurs, the material constants, as well as the natural frequencies of the plate, will decrease accordingly. Here, for illustration, the frequency “sensitivity analysis” will be performed to study how the reduction in each material constant affects the first five natural frequencies of a square [0^o^_T_] composite cantilever plate with thickness T = 200 mm, zero (0^o^) fiber angle, *a* = 1 m, aspect ratio *a*/*h* = 5, and the original material properties of *E*_1_ = 112 GPa, *E*_2_ = 11 GPa, *ν*_12_ = 0.25, *G*_12_ = *G*_13_ = 4.48 GPa, *G*_23_ = 1.5 GPa, and *ρ* = 1500 kg/m^3^. The original first five natural frequencies of the plate are *f*_1_ = 227.86, *f*_2_ = 272.99, *f*_3_ = 544.09, *f*_4_ = 845.04, and *f*_5_ = 918.32 Hz. Here, the frequency sensitivity analysis of the [0^o^_T_] composite cantilever plate is performed to study how a 20% magnitude reduction in an individual material constant can affect the natural frequencies of the plate while the other material constants remain unchanged. In the sensitivity analysis, the percentage changes of the natural frequencies are obtained to provide information about the “relative sensitivities” of the material constants on the natural frequencies. The percentage changes of the first five natural frequencies induced by the 20% reductions in the material constants obtained in different frequency sensitivity analyses are listed in Table 1.

It is noted that the results tabulated in Table 1 provide useful information about the relative sensitivities of the material constants on the natural frequencies. For instance, the first natural frequency of the plate is highly sensitive to *E*_1_ but insensitive to *G*_23_. As will be shown in the following section, the relative sensitivity information on the natural frequencies will be used in the proposed multi-level optimization method for the identification of the material constants of the plate. Next, the lamination arrangement of the [0^o^_T_] plate is changed to [0^o^_t_, 90^o^_2t_, 0^o^_t_]_s_ with t = T/8. The original natural frequencies of the square [0^o^_t_, 90^o^_2t_, 0^o^_t_]_s_ cantilever plate are *f*_1_ = 178.69, *f*_2_ = 225.06, *f*_3_ = 652.50, *f*_4_ = 713.54, and *f*_5_ = 782.80 Hz. The percentage changes of the first five natural frequencies induced by the 20% magnitude reductions in the material constants obtained in different frequency sensitivity analyses are listed in Table 2.

Similarly, the third example is the frequency sensitivity analysis of the square [45^o^_t_, −45^o^_2t_, 45^o^_t_]_s_ cantilever plate. The original natural frequencies are *f*_1_ = 121.18, *f*_2_ = 318.43, *f*_3_ = 490.72, *f*_4_ = 751.23, and *f*_5_ = 786.97 Hz. The percentage changes of the first five natural frequencies induced by the 20% magnitude reductions in the material constants obtained in different frequency sensitivity analyses are listed in Table 3.

## 4. Feasibility Study of the Sensitivity-Based Multi-Level Optimization Method

The results obtained in the above frequency sensitivity analyses of the composite cantilever plates will be used to study the feasibility and accuracy of the proposed sensitivity-based multi-level optimization method for material constants identification. The accuracy validation of the proposed method requires that both the measured natural frequencies and material constants for a composite plate be given. Herein, the natural frequencies of a plate determined using the given material constants are treated as the “measured natural frequencies”, which are then used in the proposed method to identify the material constants via a reverse engineering approach. First, consider the identification of the material constants for the above cantilever square composite [0^o^_T_] plate. The material constants with a 20% magnitude reductions are used to determine the measured natural frequencies *f*_i_* (i = 1,…, 5), i.e., *f*_1_* = 203.68, *f*_2_* = 241.28, *f*_3_* = 527.85, *f*_4_* = 760.33, and *f*_5_* = 819.79 Hz. In view of the percentage changes in Table 1, it is noted that among all the material constants, *E*_1_ and *G*_12_ have comparatively high degrees of sensitivity on natural frequencies *f*_1_ and *f*_4_. Hence, in the first level of optimization, the original values of *E_2_*, *G*_23_, and *v*_12_, the measured natural frequencies *f*_1_* and *f*_4_*, and the bounds on the design variables *E*_1_ and *G*_12_, i.e., 56 GPa < *E*_1_ < 168 GPa and 2.24 GPa < *G*_12_ < 6.72 GPa are adopted in the material constants identification process. Five pairs of (*E*_1_, *G*_12_) are randomly generated to give five starting points. It is noted that the starting points are significantly far away from the global minimum. Regarding the search direction, the parameters *LM_1_* = −7.39, *LM_3_* = −7.48, *LM_max_* = −7.48, *GDW_1_* = 1.013, and *GDW_3_* = 1 have been used to calculate the modified gradients. The five randomly generated starting points, together with their associated lowest local minima, are listed in Table 4. It is noted that, as expected, all the lowest local minima attained in the optimization are very close to the global minimum. The statistics such as average values, standard deviations, and COVs of the estimated *E*_1_ and *G*_12_ are listed in the table.

Again, in view of the percentage changes in Table 1, it is noted that regardless of *E*_1_ and *G*_12_, the natural frequencies *f*_2_, *f*_3_, and *f*_5_ are relatively sensitive to *E*_2_ and *G*_23_. Hence, in the second level of optimization, the expected values of *E*_1_ and *G*_12_ as listed in Table 4, the original value of *v*_12_, the experimental natural frequencies *f*_2_*, *f*_3_*, and *f*_5_*, and the bounds on the design variables *E*_2_ and *G*_23_, i.e., 5.5 GPa < *E*_2_ < 16.5 GPa and 0.75 GPa < *G*_23_ < 2.25 GPa are adopted in the material constants identification process. Again, five pairs of (*E*_2_, *G*_23_) are randomly generated to give five starting points. It is noted that the starting points are significantly far away from the global minimum. Regarding the search direction, the parameters *LM*_2_ = −4.59, *LM*_4_ = −3.71, *LM_max_* = −4.59, *GDW*_2_ = 1, and *GDW*_4_ = 1.237 have been used to calculate the modified gradients. The five randomly generated starting points together with their associated lowest local minima are listed in Table 5. It is noted that, as expected, all the lowest local minima attained in the optimization are very close to the global minimum. The statistics of the estimated *E*_2_ and *G*_23_ are also listed in the table.

Again, in view of the percentage changes in Table 1, it is noted that regardless of *E*_1_, *G*_12_, *E*_2_, and *G*_23_, *v*_12_ has comparatively high degrees of sensitivity on natural frequencies *f*_1_ and *f*_5_. Hence, in the third level of optimization, the average values of *E*_1_ and *G*_12_ in Table 4, the average values of *E*_2_ and *G*_23_ in Table 5, the experimental natural frequencies *f*_1_* and *f*_5_*, and the bounds on the design variable *v*_12_, i.e., 0.125 < *v*_12_ < 0.375 are adopted in the material constants identification process. First, five Poisson’s ratios are randomly generated to give five starting points. It is noted that the starting points are significantly far away from the global minimum. The randomly generated starting points together with their associated lowest local minima are listed in Table 6. It is noted that almost all the lowest local minima attained in the optimization are the same as the global minimum. The statistics of the estimated *v*_12_ are also listed in the table.

The average values and COVs of all the material constants obtained in the above three level-wise optimizations are listed in Table 7 in comparison with the true values of the material constants. It is noted that the differences between the average and the true values of the material constants are less than or equal to 0.57%. Hence, the average values can be treated as the best estimates of the material constants. It is noted that the COV of an estimate of the true material constant is a good parameter for assessing the accuracy of the estimation. In general, a COV with value less than 1% can produce a good estimate of the true material constant. Here, for comparison purpose, the stochastic global optimization method (SGOM) [58], the optimization module (Nelder–Mead Simplex Method) of Matlab (Version 2021a) [59], and the optimization module (NLPQL) of ANSYS are also used to identify all the material constants via a direct solution of the optimization problem stated in Equation (1). For each optimization method, the number of starting points adopted, the number of eigenvalue problems that have been solved, and the material constants identified in the optimization are listed in Table 8. Regarding computational efficiency, the total number of eigenvalue problems needed to be solved is the major factor that can affect the computational time, i.e., the solution of a larger number of eigenvalue problems requires longer computational time. Hence, a reduction in the number of eigenvalue problems needed to be solved can shorten the computational time and thus increase the computational efficiency. It is noted that the need for solving more eigenvalue problems requires longer computational time. Hence, among the adopted optimization methods, only the present method can identify the material constants in an accurate and efficient way.

Next, consider the material constants identification of the cantilever square composite [0^o^_t_,90^o^_2t_,0^o^_t_]_s_ plate with layer thickness t = 25 mm. After adopting the 20% reductions in the material constants, the first five “measured” natural frequencies become *f*_1_* = 159.83, *f*_2_* = 201.30, *f*_3_* = 583.62, *f*_4_* = 638.21, and *f*_5_* = 700.17 Hz. In view of the percentage changes in Table 2, it is noted that natural frequencies *f*_1_, *f*_2_, *f*_3_, and *f*_5_ are more sensitive to *E*_1_, *G*_12_, and *G*_23_ than the other material constants. Hence, in the first level of optimization, the original values of *E_2_* and *v*_12_, the measured natural frequencies *f*_1_*, *f*_2_*, *f*_3_*, and *f*_5_*, and the bounds on the design variables *E*_1_, *G*_12_, and *G*_23_, i.e., 56.00 GPa < *E*_1_ < 168.00 GPa, 2.24 GPa < *G*_12_ < 6.72 GPa, and 0.75 GPa < *G*_23_ < 2.25 GPa are adopted in the material constants identification process. Regarding the search direction, the parameters *LM*_1_ = −6.68, *LM*_3_ = −5.73, *LM*_4_ = −1.78, *LM_max_* = −6.68, *GDW*_1_ = 1, *GDW*_3_ = 1.17, and *GDW*_4_ =3.76 have been used to calculate the modified gradients. Five randomly generated starting points, together with their associated lowest local minima, are listed in Table 9. It is noted that the statistics of the estimated *E*_1_, *G*_12_, and *G*_23_ are also listed in the table.

Again, in view of the percentage changes in Table 2, it is noted that regardless of *E*_1_, *G*_12_, and *G*_23_, the natural frequencies *f*_1_, *f*_2_, and *f*_4_ are more sensitive to *E*_2_ and *v*_12_. Hence, in the second level of optimization, the average values of *E*_1_, *G*_12_, and *G*_23_, as listed in Table 9, the experimental natural frequencies *f*_1_*, *f*_2_*, and *f*_4_*, and the bounds on the design variables *E*_2_ and *v*_12_, i.e., 5.5 GPa < *E*_2_ < 16.5 GPa and 0.125 < *v*_12_ < 0.375 are adopted in the material constants identification process. Regarding the search direction, the parameters *LM_2_* = −0.62, *LM_5_* = −0.06, *LM_max_* = −0.62, *GDW_2_* = 1, and *GDW_5_* = 9.95 have been used to calculate the modified gradients. Five randomly generated starting points, together with their associated lowest local minima, are listed in Table 10. It is noted that the statistics of the estimated *E*_2_ and *G*_23_ are also listed in the table.

The average values and COVs of the estimates of all the material constants obtained in the above two level-wise optimizations are listed in Table 11 in comparison with the true material constants. It is noted that the COVs of the estimates of *E*_1_ and *E*_2_ are higher than 1%. Therefore, a third level-wise optimization is performed to lower the COVs and improve the accuracy of *E*_1_ and *E*_2_. Herein, only *E*_1_ and *E*_2_ are treated as design variables and the first five measured natural frequencies are considered in the optimization process. In view of the standard deviations of *E*_1_ and *E*_2_, the bounds are set as 87.43 GPa < *E*_1_ < 91.66 GPa and 8.62 GPa < *E*_2_ < 8.97 GPa. Regarding the search direction, the parameters *LM*_1_ = −6.68, *LM*_2_ = −0.62, *LM_max_* = −6.68, *GDW*_1_ = 1, and *GDW*_2_ = 10.85 have been used to calculate the modified gradients. Five randomly generated starting points, together with their associated lowest local minima, are listed in Table 12. It is noted that the statistics of the estimated *E*_1_ and *E*_2_ are also listed in the table.

It is noted that the differences between the average and the true values of the material constants are less than or equal to 0.43% and COVs less than 1%. Hence, in view of the results listed in Table 11 and Table 12, the average values can be treated as the best estimates of the true material constants. For comparison purpose, the SGOM, the optimization (Nelder–Mead Simplex Method) module of Matlab, and the optimization module (NLPQL) of ANSYS are also used to identify all the material constants in a direct way. For each optimization method, the number of starting points adopted, the number of eigenvalue problems that have been solved, and the material constants identified in the optimization are listed in Table 13. Again, it is noted that among the adopted optimization methods, only the present method can identify the material constants in an accurate and efficient way.

Finally, consider the identification of the material constants for the cantilever square composite [45^o^_t_, −45^o^_2t_, 45^o^_t_]_s_ plate with t = 25 mm. After adopting the 20% reductions in the material constants, the first five “measured” natural frequencies become *f*_1_* = 108.10, *f*_2_* = 285.20, *f*_3_* = 438.84, *f*_4_* = 671.83, and *f*_5_* = 704.23 Hz. In view of the percentage changes in Table 3, it is noted that *E*_1_ and *G*_12_ have comparatively high degrees of sensitivity on natural frequencies *f*_1_, *f*_2_, and *f*_3_ among all the material constants. Hence, in the first level of optimization, the original values of *E_2_*, *G*_23_, and *v*_12_, the measured natural frequencies *f*_1_*, *f*_2_*, and *f*_3_*, and the bounds on the design variables *E*_1_ and *G*_12_, i.e., 56.00 GPa < *E*_1_ < 168.00 GPa and 2.24 GPa < *G*_12_ < 6.72 GPa, are adopted in the material constants identification process. Regarding the search direction, the parameters *LM*_1_ = −7.44, *LM*_3_ = −6.66, *LM_max_* = −7.44, *GDW*_1_ = 1, and *GDW*_3_ = 1.12 have been used to calculate the modified gradients. Five randomly generated starting points, together with their associated lowest local minima, are listed in Table 14. It is noted that the statistics of the estimated *E*_1_ and *G*_12_ are also listed in the table.

Again, in view of the percentage changes in Table 3, it is noted that regardless of *E*_1_ and *G*_12_, *E*_2_, *G*_23_, and *v*_12_ have comparatively high degrees of sensitivity on natural frequencies *f*_1_, *f*_2_, *f*_4_, and *f*_5_. Hence, in the second level of optimization, the average values of *E*_1_ and *G*_12_, as listed in Table 9, the experimental natural frequencies *f*_1_*, *f*_2_*, *f*_4_*, and *f*_5_*, and the bounds on the design variables *E*_2_, *G*_23_, and *v*_12_, i.e., 5.5 GPa < *E*_2_ < 16.5 GPa, 0.75 GPa < *G*_23_ < 2.25 GPa, and 0.125 < *v*_12_ < 0.375 are adopted in the material constants identification process. Regarding the search direction, the parameters *LM*_2_ = −0.79, *LM*_4_ = −1.59, *LM*_5_ = −0.29, *LM_max_* = −1.59, *GDW*_2_ = 2.023, *GDW*_4_ = 1, and *GDW*_5_ = 5.482 have been used to calculate the modified gradients. Five randomly generated starting points, together with their associated lowest local minima, are listed in Table 15. It is noted that the statistics of the estimated *E*_2_, *G*_23_, and *v*_12_ are also listed in the table.

The average values and COVs of the estimates of all the material constants obtained in the above two level-wise optimizations are listed in Table 16 in comparison with the true material constants. It is noted that the COVs of the estimates of *E*_1_, *E*_2_, *G*_12_, and *G*_23_ are higher than 1%. Therefore, a third level-wise optimization is performed to lower the COVs and improve the accuracy of *E*_1_, *E*_2_, *G*_12_, and *G*_23_. Herein, *v*_12_ is set as 0.20, the rest of the material constants are treated as design variables, and the first five measured natural frequencies are considered in the optimization process. In view of the standard deviations of the estimates of the material constants, the bounds on the material constants are set as 85.03 GPa < *E*_1_ < 92.61 GPa, 7.89 GPa < *E*_2_ < 9.88 GPa, 3.43 GPa < *G*_12_ < 3.61 GPa, and 1.19 GPa < *G*_23_ < 1.25 GPa. Regarding the search direction, the parameters *LM_1_* = −7.44, *LM_2_* = −0.79, *LM_3_* = −6.66, *LM_4_* = −1.59, *LM_max_* = −7.44, *GDW_1_* = 1, *GDW_2_* = 9.466, *GDW_3_* = 1.117, and *GDW_4_* = 4.680 have been used to calculate the modified gradients. Five randomly generated starting points, together with their associated lowest local minima, are listed in Table 17. It is noted that the statistics of the estimated *E*_1_, *E*_2_, *G*_12_, and *G*_23_ are also listed in the table.

It is noted that the differences between the average and the true values of the material constants are less than or equal to 2.18% and COVs less than 1%. Hence, the average values can be treated as the best estimates of the true material constants. It is noted that if smaller COV for *E*_2_ is desired, an additional level-wise optimization in which only *E*_2_ is treated as design variable can be performed to attain more accurate estimate of *E*_2_. For comparison purpose, the SGOM, the genetic optimization (Nelder-Mead Simplex Method) module of Matlab, and the optimization module (NLPQL) of ANSYS are also used to identify all the material constants in a direct way. For each optimization method, the number of starting points adopted, the number of eigenvalue problems that have been solved, and the material constants identified in the optimization are listed in Table 18. Again, it is noted that among the adopted optimization methods, only the present method can identify the material constants in an accurate and efficient way.

## 5. Experimental Verification

**Figure 2:**
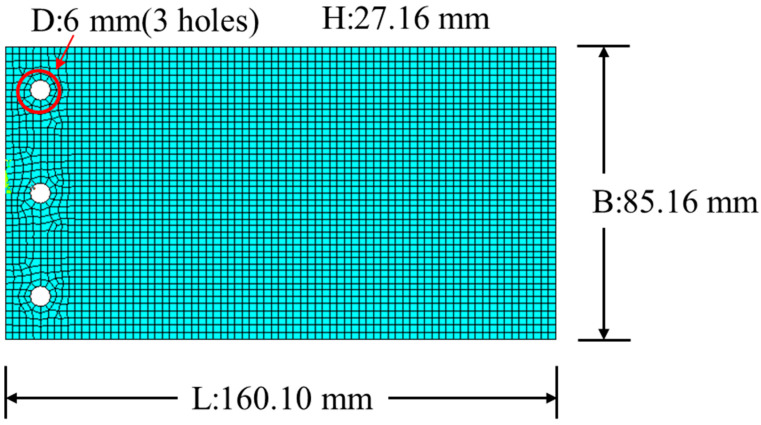
Configuration of the composite plate with the free-edge boundary condition.

**Figure 3:**
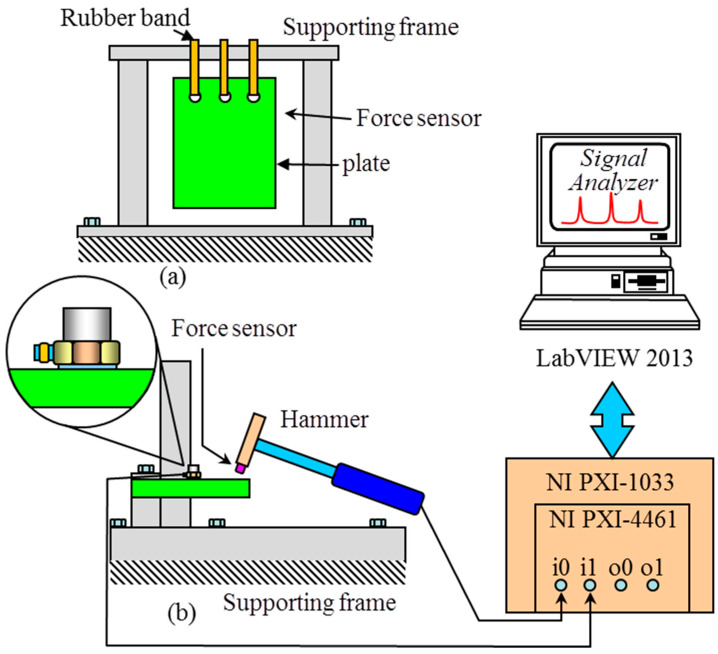
Free vibration testing of composite plate with different boundary conditions: (**a**) free-edge and (**b**) cantilever.

The experimental natural frequencies of a carbon/epoxy composite plate tested under two different boundary conditions, i.e., free-edge and cantilever, will be used to demonstrate the applicability of the proposed material constants identification method. For the case of the free-edge boundary condition, the configuration of the composite plate with *a* = 159.09 mm, *b* = 84.50 mm, *h* = 27.16 mm, and density *ρ* = 1570.47 kg/m^3^ is shown schematically in Figure 2. The [0^o^*_h_*] plate with three holes was fabricated using 332 layers of carbon/epoxy laminate with fibers orienting in the direction parallel to the long plate edge of length *a.* Rubber bands passing through the three holes were used to hand the composite plate for free vibration testing. Regarding the case of the cantilever boundary condition, the length of the free-edge composite plate became *a* = 139.96 mm and the left short edge of the [0^o^*_h_*] plate was clamped. Here, the aspect ratio (*a*/*h*) of the cantilever plate is 5.15. The experimental setup for free vibration testing of the composite plates with different boundary conditions is shown schematically in Figure 3. During the vibration test, a hammer was used to strike the plate at different points while a small accelerometer of 0.6 g was placed at a specific location to pick up the plate vibration signals. The first five mode shapes of the composite plate were used to determine the striking points and accelerometer locations on the plate. To measure a particular natural frequency, the striking points and the accelerometer locations were chosen to be away from the regions neighboring the estimated nodal lines of the associated mode shape. At least four striking points and one accelerometer location were used to measure the vibration responses of the plate. The frequency response spectra constructed from the vibration response signals were then used to extract the natural frequencies of the plate. The average values of the measured first five natural frequencies (*f_i_*, *i* = 1,…, 5) for the cantilever plate were f_1_* = 1460, f_2_* = 2013, f_3_* = 5610, f_4_* = 5726, and f_5_* = 6310 Hz with COVs less than 1.3%. On the other hand, the average values of the first two natural frequencies for the plate with free edges were f*_1_ = 2261 and f*_2_ = 2470 Hz with COVs less than 1.8%.

For comparison, several standard carbon/epoxy specimens with a thickness of 0.082 mm were also fabricated using the same fabrication process for the thick plate, with *a*/*h* = 5.15. The experimental material constants of the standard specimens were *E*_1_ = 120.6 GPa, *E*_2_ = 5.05 GPa, *ν*_12_ = 0.37, *G*_12_ = *G*_13_ = 3.9 GPa, and *G*_23_ = 1.35 GPa. Here, the material constants of the standard specimen are treated as the original material constants for the thick plate. First, the frequency sensitivity analysis of the carbon/epoxy cantilever plate is performed via the 20% material constant reduction approach. The original material constants are used in the sensitivity analysis to determine the percentage changes of the natural frequencies as listed in Table 19. The percentage changes of the first five natural frequencies listed in Table 19, together with the first five measured natural frequencies, will be used in the proposed multi-level optimization method to identify the actual material constants of the [0^o^*_h_*] plate.

The material constants identification process is exactly the same as that for the above square [0^o^_T_] cantilever plate. In view of the percentage changes in Table 19, it is noted that *E*_1_ and *G*_12_ have comparatively high degrees of sensitivity on natural frequencies *f*_1_ and *f*_4_ among all the material constants. Hence, in the first level-wise optimization, only the design variables *E*_1_ and *G*_12_ are adopted in the material constants identification process to find the statistics of *E*_1_ and *G*_12_. Again, in view of the percentage changes in Table 19, it is noted that regardless of *E*_1_ and *G*_12_, *E*_2_ and *G*_23_ have comparatively high degrees of sensitivity on natural frequencies *f*_2_, *f*_3_, and *f*_5_. Hence, in the second level-wise optimization, only the design variables *E*_2_ and *G*_23_ are adopted in the material constants identification process to find the statistics of *E*_1_ and *G*_12_. Finally, in view of the percentage changes in Table 19, it is noted that regardless of *E*_1_, *G*_12_, *E*_2_, and *G*_23_, *v*_12_ has comparatively high degrees of sensitivities on natural frequencies *f*_1_ and *f*_5_. Hence, in the third level-wise optimization, only the design variable *v*_12_ is adopted in the material constants identification process to find the statistics of *v*_12_. In summary, the statistics of the estimates of the material constants are listed in Table 20. It is noted that the COVs of the estimates of the material constants are less than 1%. Hence, the average values can be treated as the actual material constants.

It is noted that the identified material constants of the [0^o^*_h_*] cantilever plate are different from those of the standard specimen. The correctness of the identified material constants for the [0^o^*_h_*] cantilever plate will be verified using the first two measured natural frequencies of the same [0^o^*_h_*] plate with free edges. The identified material constants are then used in the finite element code of ANSYS to predict the first two natural frequencies of the free-edged [0^o^*_h_*] plate. In the finite element analysis, the 3D Solid186 elements of ANSYS, with the mesh shown in Figure 2, are adopted to predict the natural frequencies. The natural frequencies predicted using the original and identified material constants are listed in Table 21 in comparison with the experimental ones. It is noted the use of the identified material frequencies can produce a good prediction of the natural frequencies with error less than or equal to 0.15%. Finally, it is worth pointing out that the idea of the proposed sensitivity-based multi-level optimization method can be easily extended to the system parameters identification of structures using different types of measured structural responses.

## 6. Conclusions

A simple yet effective natural frequency sensitivity-based multi-level optimization method has been presented for the identification of the material constants of composite plates. The sensitivity information about the effects of material constants on the natural frequencies of a composite plate has been used to convert the complex material constants identification problem to several simple level-wise optimization problems. The statistics (expected values and coefficients of variation) of the estimates of the material constants have been obtained via the solutions of the level-wise optimization problems. An appropriate criterion based on the coefficient of variation has been established to identify the material constants. In comparison with three conventional optimization methods, it has been shown that the proposed method is able to identify the material constants of composite plates with different lamination arrangements in a more accurate and efficient way. Furthermore, it has also been shown that the through thickness shear modulus of each composite plates, which cannot be easily determined using conventional identification techniques, has been accurately and easily identified. The applications of the proposed method have been illustrated via the material constants identification of a cantilever carbon/epoxy composite plate. It has been shown that the material constants identified from the composite cantilever plate can be used to predict accurate natural frequencies for the plate with the free boundary condition. The idea of the proposed sensitivity-based multi-level optimization method may be extended to solve any system parameters identification problem using different types of measured structural responses.

## Figures and Tables

**Table 1:** Percentage change of the natural frequency induced by a 20% magnitude reduction in material constant (square [0^o^_T_] cantilever plate with *a*/*h* = 5).

Material Constant	Natural Frequency Number				
	1	2	3	4	5
	Percentage Change (%)				
*E*_1_	−7.39	−6.26	−0.98	−3.58	−2.49
*E*_2_	−0.03	−0.06	−4.59	−0.01	−1.50
*G*_12_	−3.94	−3.06	−1.86	−7.48	−5.71
*G*_23_	0.00	−1.25	−3.39	0.00	−3.71
*ν*_12_	−0.06	−0.05	−0.01	−0.03	−0.10

**Table 2:** Percentage change of the natural frequency induced by a 20% magnitude reduction in material constant (square [0^o^_t_, 90^o^_2t_, 0^o^_t_]_s_ cantilever plate with *a*/*h* = 5).

Material Constant	Natural Frequency Number				
	1	2	3	4	5
	Percentage Change (%)				
*E*_1_	−6.68	−6.68	−3.26	−4.72	−3.20
*E*_2_	−0.44	−0.46	−0.20	−0.62	−0.21
*G*_12_	−2.99	−3.01	−5.54	−4.32	−5.73
*G*_23_	−0.92	−0.40	−1.78	−1.29	−1.75
*v*_12_	−0.06	−0.06	−0.01	−0.04	−0.03

**Table 3:** Percentage change of the natural frequency induced by a 20% magnitude reduction in material constant (Square [45^o^_t_, −45^o^_2t_, 45^o^_t_]_s_ plate with *a*/*h* = 5).

Material Constant	Natural Frequency Number				
	1	2	3	4	5
	Percentage Change (%)				
*E*_1_	−5.63	−7.44	−2.17	−3.58	−3.92
*E*_2_	−0.79	−0.58	−0.25	−0.41	−0.27
*G*_12_	−4.03	−2.76	−6.66	−5.36	−5.59
*G*_23_	−0.45	−0.74	−1.27	−1.33	−1.59
*v*_12_	−0.29	0.09	−0.02	−0.01	0.01

**Table 4:** Material constants (*E*_1_ and *G*_12_) identified in the first-level optimization (square [0^o^_T_] cantilever plate with *a*/*h* = 5).

	*E*_1_ (GPa)	*G*_12_ = *G*_13_ (GPa)
True value	89.60	3.58
Starting point (*E*_1_, *G*_12_)	Identified value	
First (165.08, 2.64)	89.60	3.60
Second (117.46, 3.39)	89.60	3.60
Third (132.84, 2.94)	90.14	3.58
Fourth (103.06, 5.11)	89.82	3.58
Fivth (96.54, 6.37)	89.24	3.58
Average	89.68 (0.09) *	3.59 (0.16)
Standard deviation	0.43238	0.00026
COV (%)	0.48	0.01

* Error (%) = (|True value − identified value|/True value) × 100%.

**Table 5:** Material constants (*E*_2_ and *G*_23_) identified in the second-level optimization (square [0^o^_T_] cantilever plate with *a*/*h* = 5).

	*E*_2_ (GPa)	*G*_23_ (GPa)
True value	8.80	1.20
Starting point (*E*_2_, *G*_23_)	Identified value	
First (11.02, 1.01)	8.80	1.20
Second (13.72, 1.54)	8.80	1.20
Third (5.79, 2.17)	8.91	1.19
Fourth (7.77, 2.23)	8.80	1.20
Fifth (12.92, 1.54)	8.94	1.21
Average	8.85 (0.57) *	1.20 (0.00)
Standard deviation	0.01977	0.00028
COV (%)	0.22	0.02

* Error (%) = (|True value − identified value|/True value) × 100%.

**Table 6:** Poisson’s ratio (*v*_12_) identified in the third-level optimization (square [0^o^_T_] cantilever plate with *a*/*h* = 5).

	*ν*_12_
True value	0.20
Starting point (*ν*_12_)	Identified value
First (0.37)	0.20
Second (0.15)	0.20
Third (0.23)	0.21
Fourth (0.29)	0.20
Fifth (0.14)	0.20
Average	0.20 (0.00) *
Standard deviation	0.00023
COV (%)	0.12

* Error (%) = (|True value − identified value|/True value) × 100%.

**Table 7:** Summary of the results obtained in the three level-wise optimizations (square [0^o^_T_] cantilever plate with *a*/*h* = 5).

Level no.	Design Variable	True Value	Average	COV (%)
First	*E*_1_ (GPa)	89.60	89.68 (0.09) *	0.48
	*G*_12_ (GPa)	3.58	3.59 (0.16)	0.01
Second	*E*_2_ (GPa)	8.80	8.85 (0.57)	0.22
	*G*_23_ (GPa)	1.20	1.20 (0.00)	0.02
Third	*v*_12_	0.20	0.20 (0.00)	0.12

* Error (%) = (|True value − identified value|/True value) × 100%.

**Table 8:** Identified material constants using different optimization techniques (square [0^o^_T_] cantilever plate with *a*/*h* = 5).

	Material Constant						
	*E*_1_ (GPa)	*E*_2_ (GPa)	*G*_12_ (GPa)	*G*_23_ (GPa)	*v*_12_		
True value	89.60	8.80	3.58	1.20	0.20		
Method						Number of starting points	Number of Eigenvalue problems
Present	89.68 (0.09) *	8.85 (0.57)	3.59 (0.16)	1.20 (0.00)	0.20 (0.00)	15	150
SGOM	89.53 (0.08)	8.83 (0.37)	3.58 (0.00)	1.19 (0.57)	0.24 (22.16)	20	340
Matlab	88.37 (0.68)	8.88 (0.94)	3.58 (0.00)	1.48 (23.28)	0.31 (54.24)	1	281
ANSYS	89.86 (0.29)	8.86 (0.70)	3.58 (0.00)	1.18 (1.33)	0.13 (37.25)	1	155

* Error (%) = (|True value − identified value|/True value) × 100%.

**Table 9:** Material constants (*E*_1_, *G*_12_, *G*_23_) identified in the first-level optimization (square [0^o^_t_, 90^o^_2t_, 0^o^_t_]_s_ cantilever plate with *a*/*h* = 5).

	*E*_1_ (GPa)	*G*_12_ = *G*_13_ (GPa)	*G*_23_ (GPa)
True value	89.60	3.58	1.20
Starting point (*E*_1_, *G*_12_, *G*_23_)	Identified value		
First (57.98, 2.30, 0.99)	89.48	3.60	1.20
Second (126.78, 6.67, 1.36)	89.54	3.60	1.20
Third (81.40, 6.17, 2.03)	89.63	3.58	1.22
Fourth (116.09, 6.49, 2.01)	89.61	3.58	1.20
Fivth (91.81, 2.53, 1.22)	89.58	3.58	1.22
Average	89.17 (0.09) *	3.59 (0.16)	1.21 (0.56)
Standard deviation	1.05834	0.00224	0.00060
COV(%)	1.19	0.06	0.05

* Error (%) = (|True value − identified value|/True value) × 100%.

**Table 10:** Material constants (*E*_2_, *v_12_*) identified in the second-level optimization (square [0^o^_t_,90^o^_2t_,0^o^_t_]_s_ cantilever plate with *a*/*h* = 5).

	*E*_2_ (GPa)	*ν*_12_
True value	8.80	0.20
Starting point (*E*_2_, *ν*_12_)	Identified value	
First (12.76, 0.28)	9.09	0.20
Second (7.71, 0.31)	8.53	0.20
Third (14.21, 0.29)	8.89	0.20
Fourth (12.99, 0.29)	9.05	0.21
Fivth (8.14, 0.29)	8.87	0.20
Average	8.89 (0.98) *	0.20 (0.00)
Standard deviation	0.19490	0.00010
COV (%)	2.19	0.05

* Error (%) = (|True value − identified value|/True value) × 100%.

**Table 11:** Summary of the results obtained in the two level-wise optimizations (square [0^o^_t_, 90^o^_2t_, 0^o^_t_]_s_ cantilever plate with *a*/*h* = 5).

Level No.	Material Constants	True Value	Average	COV (%)
First	*E*_1_ (GPa)	89.60	89.17 (0.09) *	1.19
	*G*_12_ (GPa)	3.58	3.58 (0.00)	0.06
	*G*_23_ (GPa)	1.20	1.21 (0.00)	0.05
Second	*E*_2_ (GPa)	8.80	8.89 (0.57)	2.19
	*v*_12_	0.20	0.20 (0.00)	0.05

* Error (%) = (|True value − identified value|/True value) × 100%.

**Table 12:** Material constants (*E*_1_, *E*_2_, *G*_12_, *G*_23_, *v*_12_) identified in the third level-wise optimization (square [0^o^_t_, 90^o^_2t_, 0^o^_t_]_s_ cantilever plate with *a*/*h* = 5).

	*E*_1_ (GPa)	*E*_2_ (GPa)
True value	89.6	8.80
Starting point (*E*_1_, *E*_2_)	Identified value	
First (88.26, 9.22)	89.59	8.80
Second (88.56, 9.60)	89.57	8.80
Third (90.33, 8.66)	89.84	8.61
Fourth (91.60, 6.62)	89.67	8.80
Fivth (88.27, 6.49)	89.52	8.80
Average	89.64 (0.04) *	8.76 (0.43)
Standard deviation	0.06254	0.02754
COV (%)	0.07	0.31

* Error (%) = (|True value − identified value|/True value) × 100%.

**Table 13:** Identified material constants using different optimization techniques (square [0^o^_t_, 90^o^_2t_, 0^o^_t_]_s_ cantilever plate with *a*/*h* = 5).

	Material Constant						
	*E*_1_ (GPa)	*E*_2_ (GPa)	*G*_12_ (GPa)	*G*_23_ (GPa)	*v*_12_		
True value	89.60	8.80	3.58	1.20	0.20		
Method						Number of starting points	Number of Eigenvalue problems
Present	89.64 (0.04) *	8.76 (0.43)	3.58 (0.00)	1.20 (0.00)	0.20 (0.00)	15	150
Stochastic global optimization	89.69 (0.10)	8.69 (1.28)	3.58 (0.00)	1.20 (0.00)	0.20 (0.00)	20	340
Matlab	88.47 (1.26)	10.52 (19.53)	3.46 (3.47)	1.64 (36.76)	0.30 (47.81)	1	1004
ANSYS	89.18 (0.47)	12.43 (41.25)	3.69 (2.94)	1.14 (5.04)	0.13 (37.18)	1	127

* Error (%) = (|True value − identified value|/True value) × 100%.

**Table 14:** Material constants (*E*_1_, *G*_12_) identified in the first-level optimization (square [45^o^_t_, −45^o^_2t_, 45^o^_t_]_s_ cantilever plate with *a*/*h* = 5).

	*E*_1_ (GPa)	*G*_12_ = *G*_13_
True value	89.60	3.58
Starting point (*E*_1_, *G*_12_)	Identified value	
First (167.14, 6.15)	88.17	3.58
Second (144.60, 5.81)	87.84	3.59
Third (64.62, 6.04)	88.55	3.55
Fourth (57.31, 6.69)	89.27	3.55
Fivth (104.28, 5.39)	90.28	3.33
Average	88.82 (0.87) *	3.52 (1.76)
Standard deviation	3.79170	0.04668
COV (%)	4.27	1.33

* Error (%) = (|True value − identified value|/True value) × 100%.

**Table 15:** Material constants (*E*_2_, *G*_23_, *ν*_12_) identified in the second-level optimization (square [45^o^_t_, −45^o^_2t_, 45^o^_t_]_s_ cantilever plate with *a*/*h* = 5).

	*E*_2_ (GPa)	*G*_23_ (GPa)	*ν*_12_
True value	8.80	1.20	0.20
Starting point (*E*_2_, *G*_23_, *ν*_12_)	Identified value		
First (14.80, 1.63, 0.14)	9.61	1.27	0.21
Second (11.89, 1.51, 0.34)	8.62	1.19	0.19
Third (14.59, 1.47, 0.20)	9.16	1.27	0.21
Fourth (8.43, 1.39, 0.17)	8.36	1.13	0.20
Fivth (5.80, 1.11, 0.37)	8.67	1.24	0.20
Average	8.89 (0.97) *	1.22 (1.84)	0.20 (0.00)
Standard deviation	0.99758	0.01442	0.00015
COV(%)	11.23	1.18	0.08

* Error (%) = (|True value − identified value|/True value) × 100%.

**Table 16:** Summary of the results obtained in the two level-wise optimizations (square [45^o^_t_, −45^o^_2t_, 45^o^_t_]_s_ cantilever plate with *a*/*h* = 5).

Level No.	Material Constants	True Value	Average	COV (%)
First	*E*_1_ (GPa)	89.60	88.24 (0.87) *	4.27
	*G*_12_ (GPa)	3.58	3.52 (1.76)	1.33
Second	*E*_2_ (GPa)	8.80	8.89 (0.97)	11.23
	*G*_23_ (GPa)	1.20	1.22 (1.84)	1.18
	*v*_12_	0.20	0.20 (0.00)	0.08

* Error (%) = (|True value − identified value|/True value) × 100%.

**Table 17:** Material constants (*E*_1_, *E*_2_, *G*_12_, *G*_23_, *v*_12_) identified in the third level-wise optimization (square [45^o^_t_, −45^o^_2t_, 45^o^_t_]_s_ cantilever plate with *a*/*h* = 5).

	*E*_1_ (GPa)	*E*_2_ (GPa)	*G*_12_ = *G*_13_ (GPa)	*G*_23_(GPa)
True value	89.6	8.80	3.58	1.20
Starting point (*E*_1_, *E*_2_, *G*_12_, *G*_23_)	Identified value			
First (89.70, 9.22, 3.55)	89.70	8.89	3.56	1.20
Second (88.56, 9.60, 3.62)	89.70	8.85	3.58	1.20
Third (90.33, 8.66, 3.60)	89.70	9.12	3.56	1.21
Fourth (91.60, 6.62, 3.52)	89.68	9.05	3.59	1.19
Fivth (88.27, 6.49, 3.50)	89.66	9.05	3.57	1.20
Average	89.69 (0.10) *	8.99 (2.18)	3.57 (0.38)	1.20 (0.00)
Standard deviation	0.00099	0.05473	0.00053	0.00007
COV (%)	0.00	0.61	0.01	0.01

* Error (%) = (|True value − identified value|/True value) × 100%.

**Table 18:** Identified material constants using different optimization techniques (square [45^o^_t_, −45^o^_2t_, 45^o^_t_]_s_ cantilever plate with *a*/*h* = 5).

	Material Constant						
	*E*_1_ (GPa)	*E*_2_ (GPa)	*G*_12_ (GPa)	*G*_23_ (GPa)	*v*_12_		
True value	89.60	8.80	3.58	1.20	0.20		
Method						Number of starting points	Number of Eigenvalue problems
Present	89.69 (0.10) *	8.99 (2.18)	3.57 (0.38)	1.20 (0.00)	0.20 (0.00)	15	150
Stochastic global optimization	89.40 (0.23)	8.73 (0.78)	3.59 (0.07)	1.21 (0.56)	0.20 (0.00)	20	340
Matlab	86.11 (3.86)	11.73 (33.30)	3.43 (4.29)	1.76 (47.03)	0.28 (40.71)	1	526
ANSYS	89.13 (7.22)	15.05 (71.08)	3.56 (0.55)	1.20 (0.00)	0.13 37.19	1	144

* Error (%) = (|True value − identified value|/True value) × 100%.

**Table 19:** Percentage change of the natural frequency ([0^o^*_h_*] cantilever plate, *a*/*h* = 5.12).

Material Constant	Natural Frequency Number				
	1	2	3	4	5
	Percentage Change (%)				
*E*_1_	−7.25	−3.27	−0.58	−3.35	−2.09
*E*_2_	−0.03	−0.10	−3.67	−0.05	−0.07
*G*_12_	−4.09	−5.14	−2.50	−6.96	−6.83
*G*_23_	0.00	−2.12	−4.96	−0.05	−1.77
*ν*_12_	−0.06	−0.04	−0.01	−0.05	−0.04

**Table 20:** Statistics obtained in the level-wise optimization.

Level no.	Material Constants	Average	COV (%)
First	*E*_1_ (GPa)	77.56	0.07
	*G*_12_ (GPa)	4.12	0.05
Second	*E*_2_ (GPa)	6.37	0.08
	*G*_23_ (GPa)	1.33	0.61
Third	*v*_12_	0.372	0.01

**Table 21:** Natural frequencies of the [0^o^*_h_*] free-edged plate predicted using different material constants.

Natural Frequency (Hz)	*f*_1_	*f*_2_
Experimental	2261	2470
Theoretical (Original material constants)	2663 (17.78)	5237 (111.99)
Theoretical (Identified material constants)	2280 (0.87) *	2474 (0.15)

* Error (%) = (|Experimental − Theoretical|)/Experimental * 100%.

## Data Availability

The original contributions presented in this study are included in the article. Further inquiries can be directed to the corresponding author.
